# Chemical Analysis of Pottery Demonstrates Prehistoric Origin for High-Altitude Alpine Dairying

**DOI:** 10.1371/journal.pone.0151442

**Published:** 2016-04-21

**Authors:** Francesco Carrer, André Carlo Colonese, Alexandre Lucquin, Eduardo Petersen Guedes, Anu Thompson, Kevin Walsh, Thomas Reitmaier, Oliver E. Craig

**Affiliations:** 1 Department of Archaeology, University of York, York, United Kingdom; 2 BioArCh, Department of Archaeology, University of York, York, United Kingdom; 3 Instituto de Biociências, Universidade Federal do Rio Grande do Sul, Porto Alegre, RS, Brazil; 4 School of Environmental Sciences, The University of Liverpool, Liverpool, United Kingdom; 5 Archaeological Service of the Canton of Grisons, Chur, Switzerland; New York State Museum, UNITED STATES

## Abstract

The European high Alps are internationally renowned for their dairy produce, which are of huge cultural and economic significance to the region. Although the recent history of alpine dairying has been well studied, virtually nothing is known regarding the origins of this practice. This is due to poor preservation of high altitude archaeological sites and the ephemeral nature of transhumance economic practices. Archaeologists have suggested that stone structures that appear around 3,000 years ago are associated with more intense seasonal occupation of the high Alps and perhaps the establishment of new economic strategies. Here, we report on organic residue analysis of small fragments of pottery sherds that are occasionally preserved both at these sites and earlier prehistoric rock-shelters. Based mainly on isotopic criteria, dairy lipids could only be identified on ceramics from the stone structures, which date to the Iron Age (ca. 3,000–2,500 BP), providing the earliest evidence of this practice in the high Alps. Dairy production in such a marginal environment implies a high degree of risk even by today’s standards. We postulate that this practice was driven by population increase and climate deterioration that put pressure on lowland agropastoral systems and the establishment of more extensive trade networks, leading to greater demand for highly nutritious and transportable dairy products.

## Introduction

Today, alpine dairying is both a multi-million euro industry and an important cultural tradition [1]. The production of dairy foods in the high Alps (>1,800 m) is inherently risky. It requires a close understanding of the mountain environment [2,3], careful management of livestock and a massive input of labour with the reward of nutritious, storable produce from seasonal pasture that would otherwise be unused. The recent history of alpine dairying is well documented [4] but due to the ephemeral nature of transhumance there is frustratingly little archaeological evidence, a problem exacerbated by the high mountain acidic geologies that lead to deterioration of any faunal remains. Consequently the origins of alpine dairying are still widely debated [5,6] and very little is known regarding the cultural, economic or environmental context that lead to its establishment.

In Europe, we know from archaeological faunal assemblages and chemical evidence of dairy fats associated with pottery that milk production in lowland settings dates back to the Early Neolithic period when domesticated cattle and sheep were first introduced [7–9]. From this evidence, it has been much harder to establish the intensity or nature of dairying and its subsequent development. Early Neolithic ceramic sieves for separating curds and whey provide the strongest evidence for cheese production [10] and widespread reliance on fermented milk products is likely given that the ability to digest the sugars (lactose) in raw milk was an adaptation that probably only appeared in Europe during the Bronze Age [11]. In the circum-alpine lowlands, the earliest direct evidence of dairying comes from organic residues on pottery vessels dating to the late Neolithic of this region, ca. 6,000 BP [12,13]. Here, dairying was initially part of mixed economy that also included meat production with little evidence for specialization.

From the start of the 3^rd^ millennium BC, it has been hypothesised that dairying intensified with a range of interlinked innovations, that included greater reliance on ‘ante-mortem’ animal products such as wool and milk and the colonisation of poorer and less accessible land [14,15]. The little archaeological evidence for exploitation of high alpine environments that exists does not contradict this hypothesis. Seasonal occupation of high-altitudes intensified from the mid-3^rd^ millennium BC to the 1^st^ millennium BC (Bronze Age and Iron Age) and large dry stone structures begin to appear at this time [16–22]. These enclosures have been tentatively identified as animal corrals [16,23], but the near absence of any artefacts or animal bones means that their function is far from clear with no evidence to link these prehistoric sites to dairying. Indeed, prehistoric exploitation of such high altitude environments for dairying would seem to be remarkable given the high risk and sophisticated husbandry practices that are required, even by today’s standards.

**Fig 1:**
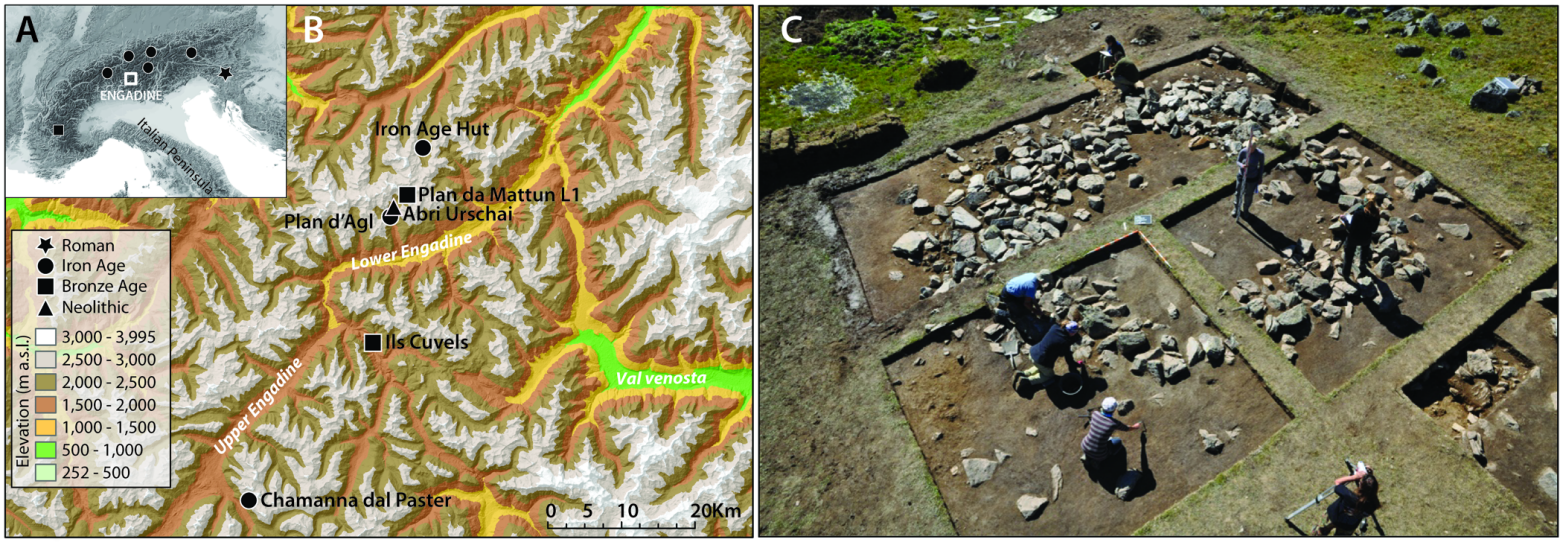
Location and chronology of the sites investigated in this study. Inset (A) location and chronology of the earliest upland dry-stone structures in the Alps with secure dates; the Iron Age Hut of Val Fenga during excavation (C).

To explore further, we provide here the first chemical evidence for the use of prehistoric pottery in this extreme environmental setting. Molecular and stable isotope analysis of lipids extracted from pottery vessels are well established techniques for discriminating dairy fats in the archaeological record [7,10,24]. Unfortunately, ceramic vessels, that have been fundamental for establishing prehistoric dairying practices elsewhere in Europe, are not routinely recovered from high altitude sites and the few potsherds that have been found are small and highly fragmented (S2 Fig), partly due to the poor preservation of these sites. Nevertheless, thirty fragments from six highland archaeological sites (Table 1) of the Engadin region of southern Switzerland (Fig 1B) were obtained from securely ^14^C dated contexts from the 5^th^ millennium BC (Neolithic) to the 1^st^ millennium BC (S1 Text). This region is typical of the central-alpine environment, with valley bottoms above 1,000 m asl and high seasonal pastures ranging from around 2,000 m to 2,800 m asl (S1 Text). Five of the sites chosen are above 2,000 m asl and include early Neolithic and Bronze Age rock-shelters and the later Iron Age stone enclosure and hut (Table 1).

**Table 1:** Main characteristics of the investigated sites and summary results of the analysis.

Site	Altitude	Date	Period	Number of potsherds analysed	Number of potsherds containing dairy fats
**Abri Urschai (Val Urschai, Ftan)**	2,180 m	5^th^ millennium BC	Neolithic	6	0
**Ils Cuvels (Ova Spin, Zernez)**	1,680 m	First half of the 2^nd^ millennium	Bronze Age	4	0
**Plan da Mattun L1 (Val Urschai, Ftan)**	2,287 m	Late 2^nd^/Early 1^st^ millennium BC	Bronze Age	6	0
**Iron Age Hut (Val Fenga, Ramosch)**	2,283 m	First half of the 1^st^ millennium BC	Iron Age	5	3
**Chamanna dal Paster (Val Languard, Pontesina)**	2,415 m	First half of the 1^st^ millennium BC	Iron Age	4	2
**Plan d’Agl (Ardez, Val Tasna)**	2,060 m	Late 2^nd^/1^st^ millennium BC	Late Bronze Age/Iron Age	5	2

## Materials and Methods

The Archaeological Service of the Canton of Grisons (Switzerland) provided permits for the archaeological investigation of the sites considered in this study (2007–2014), as well as for the analysis of 30 archaeological potsherds collected at these sites. All the samples are still available for the replication of this study. Further information on the sites and ceramic sherds is available in Supporting Information (S1 Text, S1 and S2 Tables).

Lipids were extracted and methylated in one-step with acidified methanol [25,26] in order to maximise recovery from the small samples available. Briefly, methanol (4 ml) was added to homogenized ceramic powder (1 g) drilled from the sherd surface and the mixture was sonicated for 15 min and then acidified with concentrated sulphuric acid (200 μl). The acidified suspension was heated in sealed tubes for 4 h at 70°C and then cooled, and lipids were extracted with n-hexane (3 × 2 ml) and directly analysed by GC-MS and GC-C-IRMS. GC-MS was carried out on all samples using a 7890A Series chromatograph attached to a 5975C Inert XL mass-selective detector with a quadrupole mass analyser (Agilent Technologies, Cheadle, UK). The carrier gas used was helium, and the inlet/column head-pressure was constant. A splitless injector was used and maintained at 300°C. The GC column was inserted directly into the ion source of the mass spectrometer. The ionisation energy of the mass spectrometer was 70 eV and spectra were obtained by scanning between m/z 50 and 800. Aliquots of these extracts were initially analysed using a DB-5ms (5%-phenyl)-methylpolysiloxane column (30 m × 0.250 mm × 0.25 μm; J&W Scientific, Folsom, CA, USA). The temperature for this column was set at 50°C for 2 min, then raised by 10°C min^-1^ to 325°C, where it was held for 15 min.

For GC-C-IRMS we use the instrumentation, conditions and protocols previously described in Craig et al. [24]. Instrument precision on repeated measurements was ±0.3‰ (s.e.m.) and the accuracy determined from FAME and n-alkane isotope standards was ±0.5‰ (s.e.m.). Modern reference samples were further corrected for the burning of fossil fuels to allow comparison with archaeological data. All δ^13^C values are relative to Vienna PeeDee Belemnite (VPDB) international standard. Correlations between the frequency and abundance of saturated and unsaturated FAMES were explored using PCA (variance-covariance test) in PAST 3.x [27].

Solvent extraction was undertaken where sufficient sample remained. Homogenized ceramic powders (1 g) were sonicated three times with DCM:MeOH (2:1, v/v). These total lipid extracts were combined, and evaporated to dryness under a stream of N2 and silylated with excess BSTFA + 1% TMCS at 70°C for 1 h, and then evaporated to dryness. The silylated solvent extracts were analysed by high temperature GC-MS using a DB1-HT (15 m x 0.32 mm, 0.1 mm film thickness; Agilent, UK). The temperature program was a 50°C isothermal hold followed by an increase to 350°C at 10°C min-1, followed by a 10 min isothermal hold.

## Results and Discussion

### Organic residue analysis

The amount of lipid released from these small weathered fragments of pottery were highly variable. The Iron Age pots regardless of site yielded much higher amounts (median = 434 μg g-^1^) compared to the earlier periods (median = 21 μg g-^1^, S1 Table) suggesting they may have been used differently. Analysis by gas chromatography mass spectrometry (GC-MS; S1 Table) revealed a range of saturated mid-chain length *n*-alkanoic acids (fatty acids) with even numbers of carbon atoms on most of the vessels from all periods, particularly dominated by C_16:0_ (palmitic acid) and C_18:0_ (stearic acid). These lipid profiles are typical of degraded animal fats [28] but the absence of any more diagnostic mono-, di- and triacylglycerides, assessed by solvent extraction, where sufficient sample permitted (S1 Table), prevents further identification. Several Bronze Age and Iron Age sherds contained ketones with chain lengths consistent with the heat transformation of the most abundant saturated fatty acids [29] providing the best evidence that the vessels were used for heating animal products.

To provide more specific information, 28 sherds with the most abundant C_16:0_ and C_18:0_ acids were selected for GC-combustion- isotope ratio mass spectrometry (GC-C-IRMS) with the aim of distinguishing the origins of these compounds based on their stable carbon isotope value (δ^13^C). Preferential routing of C_18:0_ from diet, following biohydrogenation of plant fatty acids in the rumen, causes a relative depletion in ^13^C relative to other fatty acids (e.g. C_16:0_) in the milk of ruminant animals and to a lesser degree in ruminant carcass fats [30]. Thus the difference between the δ^13^C values of C_16:0_ and C_18:0_ (Δ^13^C) provides an eloquent and robust way to identify dairy and carcass fats of domesticated animals.

**Fig 2:**
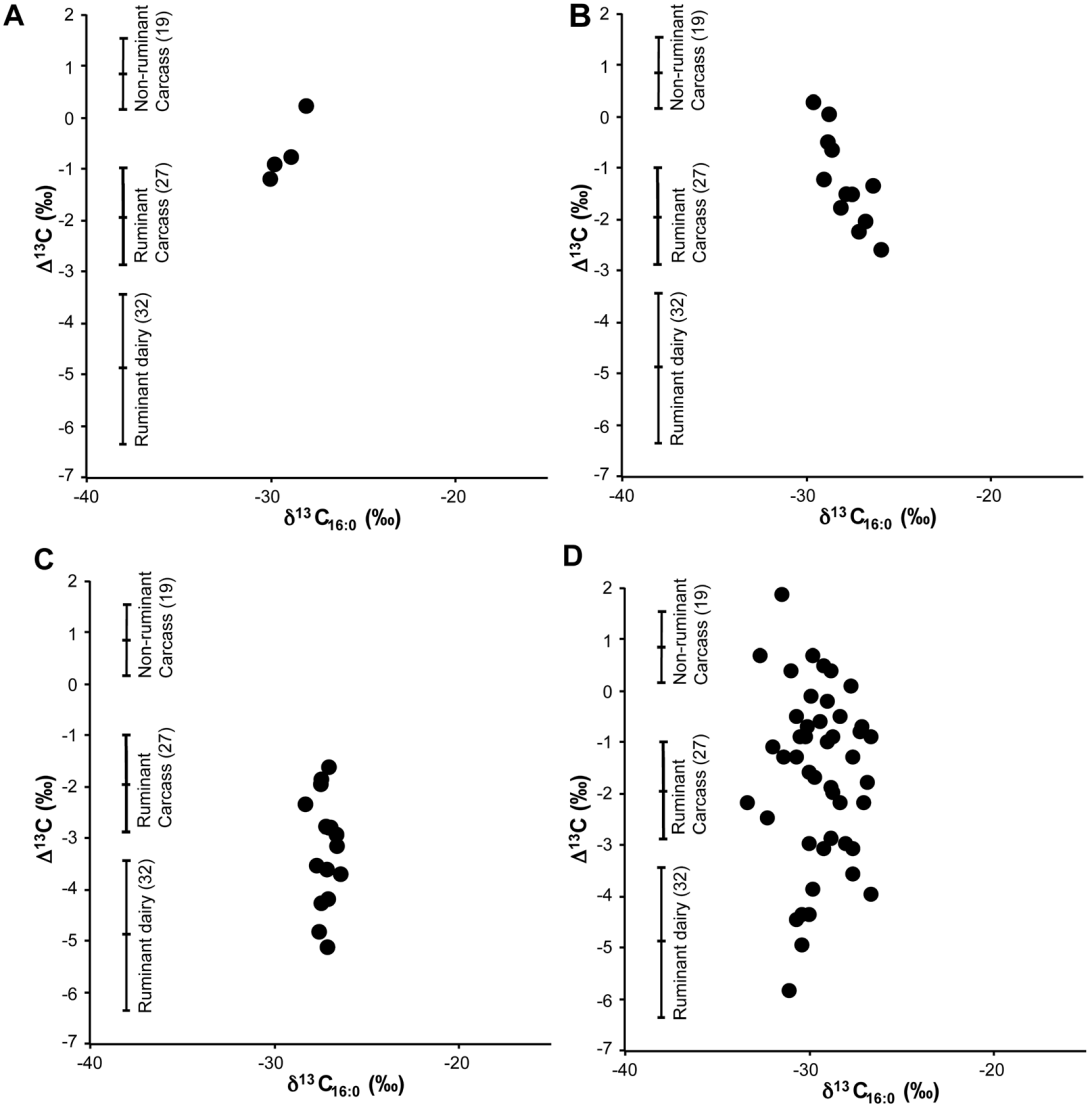
Plots of the Δ^13^C values for animal fat residues in archaeological pottery from the Alpine highland and foreland. Pottery vessels are from (A) Neolithic high-altitude alpine sites; (B) Bronze Age high-altitude alpine sites; (C) Iron Age high-altitude alpine sites; and (D) Late Neolithic lakeside settlements in the Alpine foreland [12,13]. Mean values and ranges (±1 SD) of authentic reference fats [12,31] are shown (n = number of observations) but exclude the deer samples which are likely to be absent from the high altitude sites investigated.

In Fig 2, the fatty acid δ^13^C values extracted from 28 pots are compared with 78 dairy, ruminant and non-ruminant modern authentic fats. To check for regional variation, the modern reference data set has been augmented with samples of milk, cheese and meat produced locally in the Engadin from high-pastured domesticated animals, a carcass fat sample of marmot (*Marmota marmota*) as well as several samples of wild ruminants, chamois (*Rupicapra rupicapra*), roe deer (*Capreolus capreolus*), red deer (*Cervus elaphus*) and Ibex (*Capra ibex*; S2 Table). As shown in previous studies [7], the range of Δ^13^C values for dairy products is clearly distinguishable from other ruminant and non-ruminant products. One exception is the red deer sample which produced a Δ^13^C value (-4.3‰) within the upper dairy range and is consistent with previous measurements made on the fats from this species [24].

**Fig 3:**
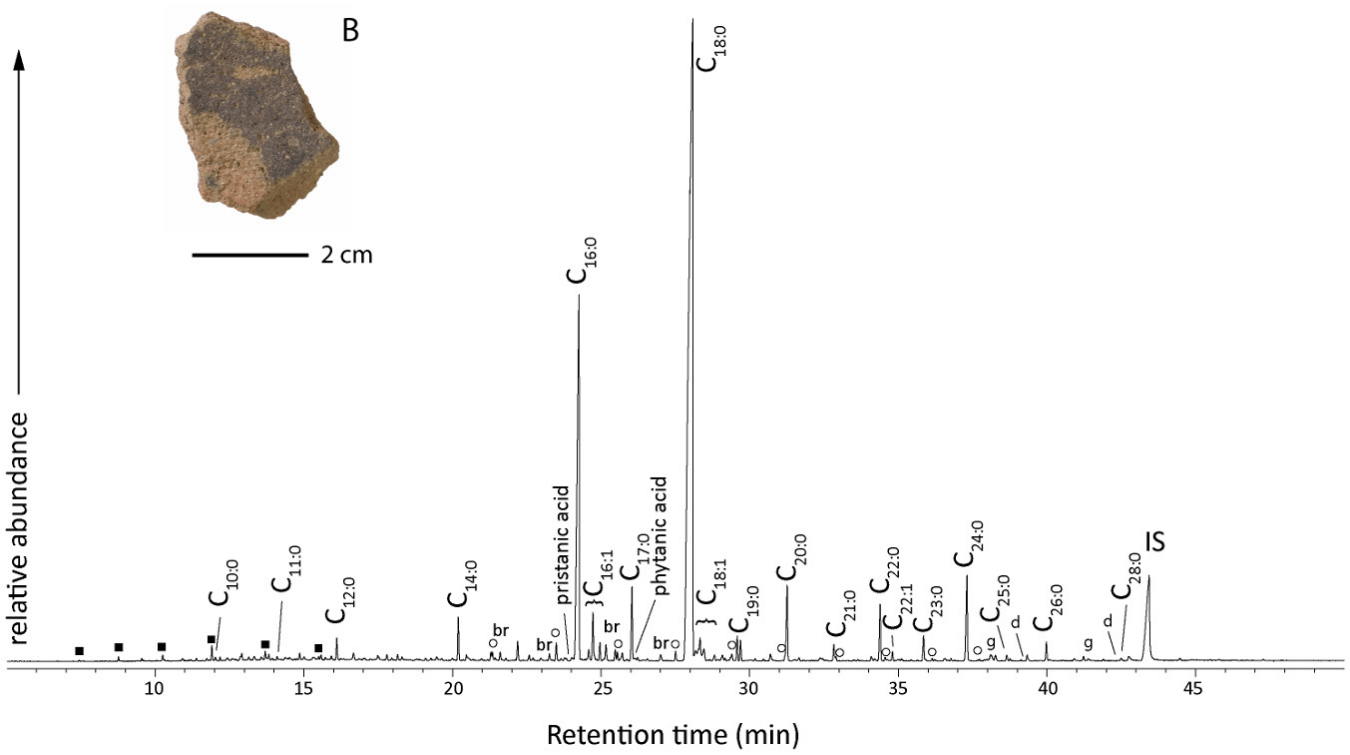
Partial gas chromatograms of an acid methanol extract from a potsherd (Val Languard, sample 27). Cn:x, fatty acids with carbon length n and number of unsaturation x; square, alkanes; circle, α,ω-dicarboxylic fatty acids; br, branched chain fatty acids; γ, γ-lactones; δ, δ-lactones. Inset (B): photo of the potsherd analysed, typical of the small fragments recovered. The sample was analysed using a DB-5ms capillary column (30 m × 0.250 mm × 0.25 μm) using the temperature program described in the text.

Seven potsherds of the 28 suitable for analysis had fatty acid Δ^13^C values within one standard deviation of the mean value for the reference dairy fats. Significantly, all of these samples dated to the Iron Age (Fig 2C), providing the earliest evidence of dairy practice at high altitude. In contrast, lipids from the earlier upland Neolithic and Bronze Age pottery had carbon isotope values consistent with ruminant and non-ruminant carcass fats, either from domesticated or wild species (Fig 2A and 2B). These patterns are distinguishable from late Neolithic lowland pottery assemblages from Swiss lakeside settlements (Fig 2D), which include dairy fats, but also ruminant and non-ruminant adipose and potentially plant derived lipids [12,13]. Although dairying had always been a feature of mixed lowland pastoral economies, the pottery evidence shows it only penetrated the upland regions during the Late Bronze Age. In total, dairy was identified at all the three of the Iron Age sites investigated. Despite some overlap in the reference ranges, it is highly unlikely that any of these residues are derived from red deer. Whilst, there is clear evidence for the presence of red deer at lowland and mid-altitude sites (<1,800 m) during the Neolithic, they were much less frequently exploited during the Bronze and Iron Ages [32]. Deer is also rarely found in upland animal bone assemblages, with no evidence on the high altitude Iron Age sites investigated here, which instead are dominated by the remains of domesticated ruminants (S1 Text). In addition, 4 of the Iron Age potsherds, identified as containing dairy products by GC-C-IRMS, group together in a principal components analysis (PCA) of fatty acid distributions (S1 Fig). Although the relative abundance of fatty acids are highly susceptible to alteration in the burial environment, these vessels retained lower molecular weight saturated fatty acids (C_8_-C_14_; Fig 3) which are at much higher relative abundance in ruminant dairy fats compared to other animal fats.

### Archaeological Significance

It is reasonable to assume that pottery was used to heat milk as part of the cheese production process; for which there are direct ethnographic examples [33]. Interestingly, the ceramics containing dairy fats are associated with stone structures (Fig 1C) suggesting that the latter were an essential part of dairy focused alpine pastoralism. Similar structures are used by modern alpine pastoralists for the production of cheese during the summer months [34,35]. As these structures are found several hundred years earlier in other parts of the Alps [16], an earlier origin for alpine dairying may be postulated but harder to demonstrate given the lack of artefacts at these sites. Nevertheless, the chronology of the earliest stone structures recorded (Fig 1A), suggests that high-altitude dairying became widespread in the central- and eastern-alpine areas during the Iron Age. By contrast, pottery from the Neolithic and Bronze Age rock-shelters contained carcass fats, which is consistent with the intermittent occupation of temporary shelters by transhumance herders [36,37], who may have supplemented their diet by hunting wild mountain animals.

Palaeoenvironmental analysis [38–40], including the recovery of ancient DNA from lake sediments [41], has only permitted the identification of the presence of stock animals in Iron Age alpine landscapes, rather than yielding specific information on economic practices. Similarly, the shift from a mixed economy, including wild hunted resources, in the Neolithic to greater reliance on domesticates and dairying in the Late Bronze Age and Iron Age is also observed in the lowland faunal assemblages [32] but cannot be used to securely infer increase dairying at this time. Indeed, the extraordinary effort to produce dairy products at high altitude, that our data implies, is unexpected. Iron Age herders must have possessed detailed knowledge of the location of pastures, they must have been able to carefully control the sex and age composition of herds and have had the technological ability to transform milk into storable and transportable dairy products in high mountain environments. As today, prehistoric alpine pastoralists would have had to overcome adverse and unpredictable weather and cope with a significant reduction in the yield and quality of milk from their mountain pastured animals [42].

There are many reasons for the development of high altitude alpine dairying at this juncture in prehistory. Pressure on the lowland pasture and an increased demand for alpine cheese provides the most parsimonious explanation. This was probably triggered by social, economic and environmental processes that took place during the Late Bronze Age and the Early Iron Age. The increasing number of settlements, especially at middle altitude [43], accounts for demographic growth, that led to the intensification of arable agriculture and husbandry [44]. The occurrence of scythes in the Alps since the Iron Age is usually associated with intensification in foddering to enable the winter stabling of bigger flocks and herds [5]. Evidence of increasing social complexity and large-scale intra- and trans-alpine contacts [45] suggests the presence of specialized crafting and farming productions and the creation of wide commercial networks. Climate deterioration, evidenced by palaeoenvironmental data, threatened certain forms of arable agriculture [46] and forced the enhancement of alternative food production strategies. Salt mining in the Eastern Alps, well documented for this period [47], might have favoured the maturing of cheese in the uplands during the summer [48], thus providing alpine communities with a high protein storable commodity that could be consumed during the winter months or traded/exchanged in the surrounding regions. We know from documented sources that alpine cheese was highly valued in the Roman period (Strabo, Geogr. IV 6, 8) and was transported widely across the Roman provinces. All of the aforementioned processes would have been situated within a complex social and ideological network. The consumption of dairy products and meat were also integral elements in feasting in an increasingly structured, hierarchical society where conspicuous consumption/possession of certain foods and material culture become increasingly important [49,50]. These inferences highlight that high-altitude dairying had as much of a key-role in the development of alpine communities during the 1st millennium BC, as it had in later periods of the history of the Alps, for which it is celebrated.

## Conclusions

The development of high altitude dairying represents a new form of niche construction: the manipulation of part of a landscape for specific economic activities [51], i.e. the production of highly nutritious resource that can be easily transported and exchanged. This strategy has contributed to managing and preserving the upland environments over time, and is currently contributing to promoting cultural and gastronomic tourism. Remarkably, these high altitude environments have sustained dairy based pastoralism for over 3,000 years.

Alpine cheese is renowned to have a long and complex history, that made it an essential feature of alpine cultural heritage [1]. This study showed that its origin can be traced back to prehistory, and that it is deeply related to the socio-economic development of alpine communities and to the transformation of upland landscapes. This research demonstrates the long-term resilience and persistence of the landscape management strategies associated with dairying activity, a form of anthropic landscape which has stood the test of time. Nowadays it is threatened by climate change and new supranational economic food production strategies that ignore, or are unaware of the complex, successful forms of local environmental knowledge and associated food production practices [52]. Therefore, the promotion of protection policies for traditional alpine cheeses and upland landscapes has to consider their long-term mutual correlation, which this study has dated back to the prehistoric period.

## Supporting Information

S1 Fig Principal component analysis (PCA) of frequency and abundance of saturated FAMES. Two main components explain comprehensively 95.2% of variance in FAMES distribution. The vast majority of the variance (Component 1; 87.5%) is associated with the relative contribution of C16:0 (left) over C18:0 (right), which could be tentatively associated with acyl lipids sourced from ruminant (C18:0) and non-ruminant (C16:0) adipose fat. The second most important source of variance is the frequency and abundance of small- and large-chain acyl lipids (Component 2; 7.7%). Notably most of the sherds containing short-chain FA (e.g. C5 to C14) have Δ^13^C values consistent with modern authentic dairy fats.
(TIF)

S2 Fig Photos of ceramic sherds containing dairying lipids. Sherd IDs correspond to those reported in S1 Table. For each sherd the left photo illustrates the exterior and the right the interior.
(TIF)

S1 Table Summary of lipid data; ceramic sherd selected for lipid analysis by GC-MS and GC-C-IRMS. (Cn) or (Cn:x)—carbon length n and number of unsaturations x, SFA—saturated fatty acid, UFA—unsaturated fatty acid, DCFA—α,ω-dicarboxylic fatty acids, br -branched chain fatty acids, APFA—ω-(o-alkylphenyl) alkanoic acids, pri.—pristanic acid, phy.—phytanic acid, Alk.—alkane, Ket.—Ketone, lact.—lactone, chol.—cholesterol derivative, abiet. - 7-Oxodehydroabietic acid and dehydroabietatic acid, terp.—unresolved terpenes mixture.
(DOCX)

S2 Table Summary of GC-C-IRMS analysis of faunal remains. (DOCX)

S3 Table Radiocarbon dates from the sites associated with pottery analysed. (DOCX)

S1 Text Geographical setting and brief description of the investigated sites. (DOCX)
