# Supplementary figures and images for: Chemical Analysis of Pottery Demonstrates Prehistoric Origin for High-Altitude Alpine Dairying
Source: PLoS One. 2016 Apr 21;11(4):e0151442. doi: 10.1371/journal.pone.0151442 (PMC4839595; doi:10.1371/journal.pone.0151442)

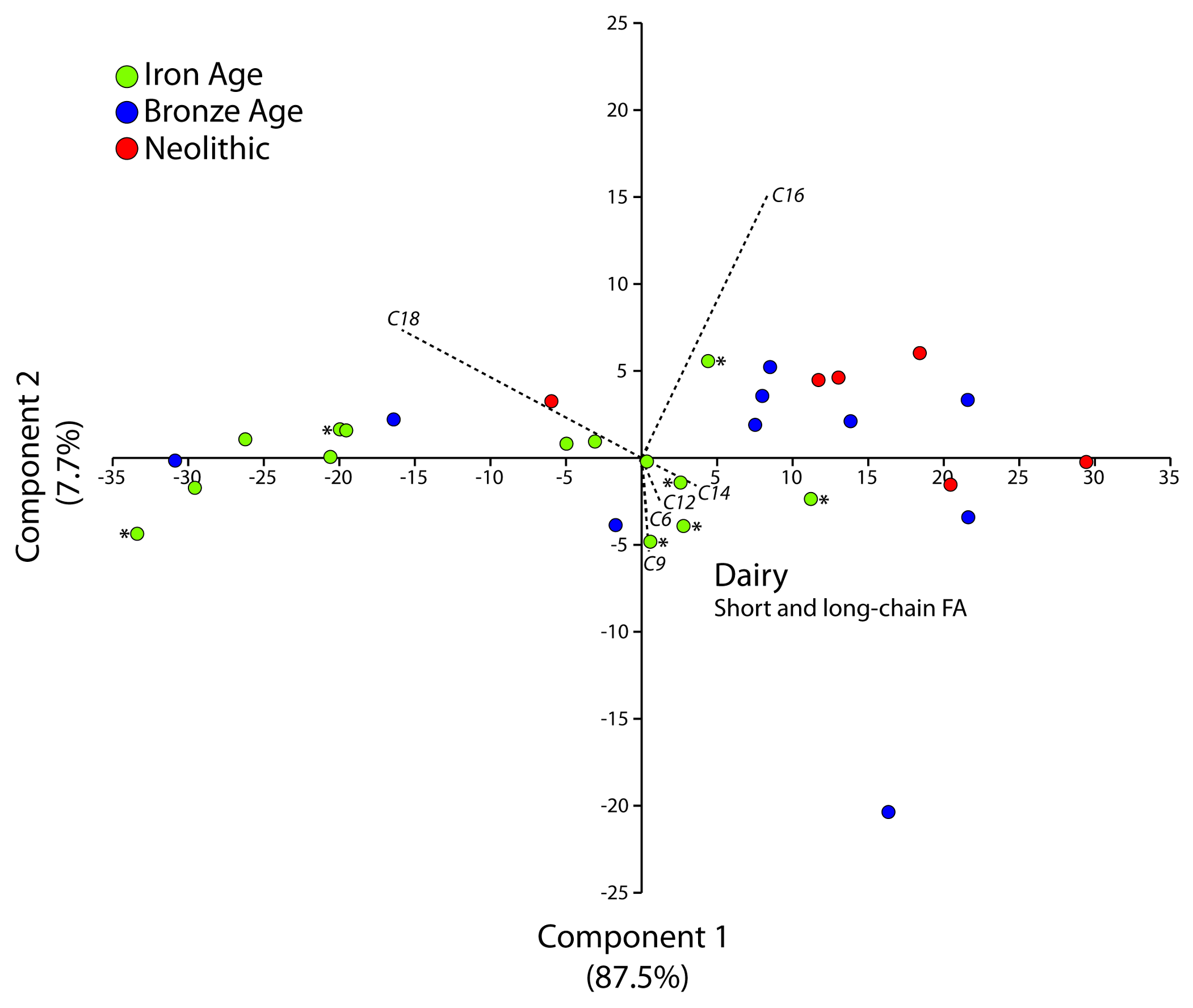

Supplement: S1 Fig — Two main components explain comprehensively 95.2% of variance in FAMES distribution. The vast majority of the variance (Component 1; 87.5%) is associated with the relative contribution of C16:0 (left) over C18:0 (right), which could be tentatively associated with acyl lipids sourced from ruminant (C18:0) and non-ruminant (C16:0) adipose fat. The second most important source of variance is the frequency and abundance of small- and large-chain acyl lipids (Component 2; 7.7%). Notably most of the sherds containing short-chain FA (e.g. C5 to C14) have Δ13C values consistent with modern authentic dairy fats. (TIF) [file pone.0151442.s001.tif]

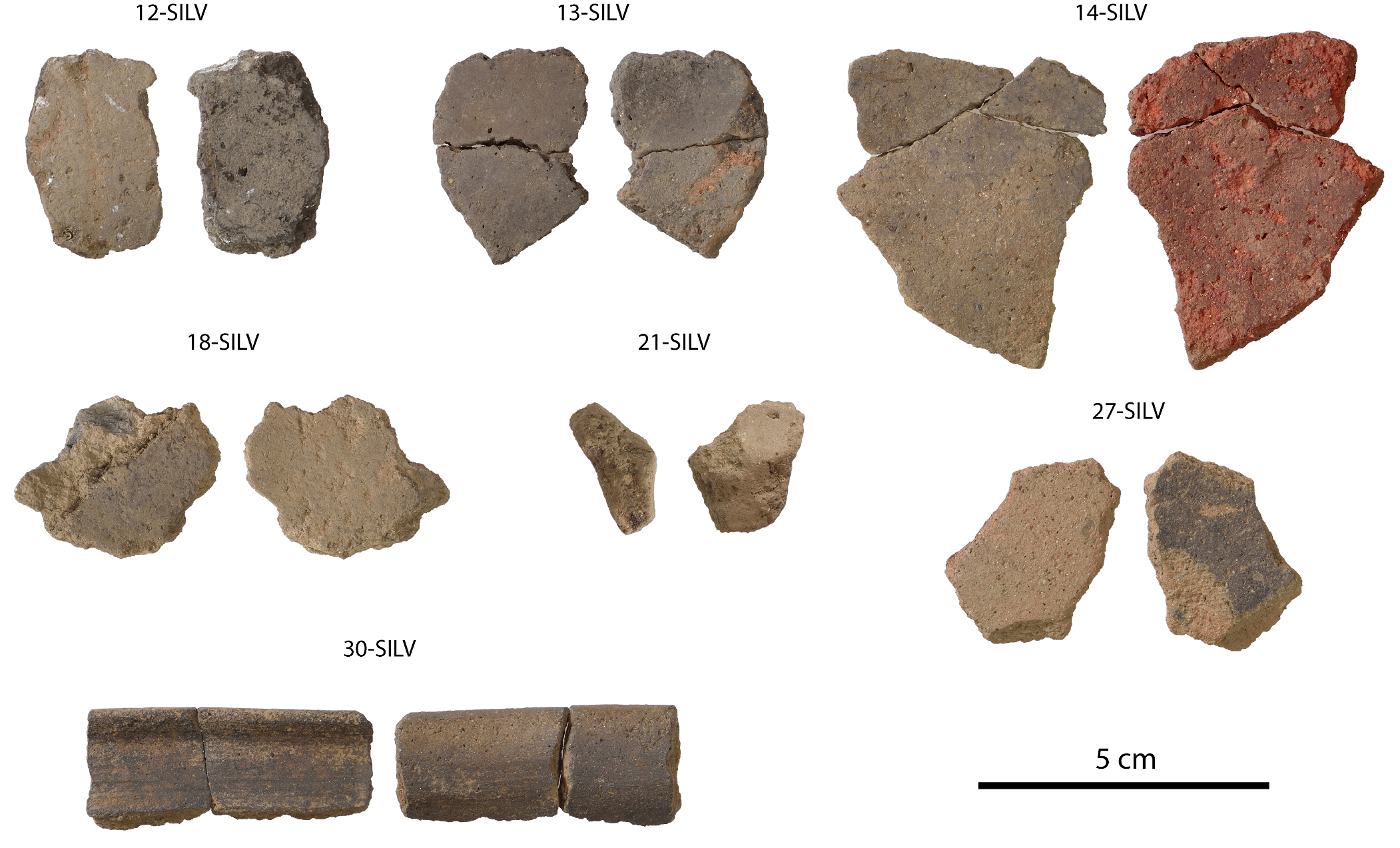

Supplement: S2 Fig — Sherd IDs correspond to those reported in S1 Table. For each sherd the left photo illustrates the exterior and the right the interior. (TIF) [file pone.0151442.s002.tif]
