# Supplementary material for: Chemical Analysis of Pottery Demonstrates Prehistoric Origin for High-Altitude Alpine Dairying
Source: PLoS One. 2016 Apr 21;11(4):e0151442. doi: 10.1371/journal.pone.0151442 (PMC4839595; doi:10.1371/journal.pone.0151442)
Supplement: S1 Table — (Cn) or (Cn:x)—carbon length n and number of unsaturations x, SFA—saturated fatty acid, UFA—unsaturated fatty acid, DCFA—α,ω-dicarboxylic fatty acids, br -branched chain fatty acids, APFA—ω-(o-alkylphenyl) alkanoic acids, pri.—pristanic acid, phy.—phytanic acid, Alk.—alkane, Ket.—Ketone, lact.—lactone, chol.—cholesterol derivative, abiet. - 7-Oxodehydroabietic acid and dehydroabietatic acid, terp.—unresolved terpenes mixture. (DOCX) [file pone.0151442.s003.docx]

**S1 Table. Summary of lipid data;** **ceramic sherd selected for lipid analysis by GC-MS and GC-C-IRMS.** (Cn) or (Cn:x) - carbon length n and number of unsaturations x, SFA - saturated fatty acid, UFA - unsaturated fatty acid, DCFA - α,ω-dicarboxylic fatty acids, br -branched chain fatty acids, APFA - ω-(o-alkylphenyl) alkanoic acids, pri. - pristanic acid, phy. - phytanic acid, Alk. - alkane, Ket. - Ketone, lact. - lactone, chol. - cholesterol derivative, abiet. - 7-Oxodehydroabietic acid and dehydroabietatic acid, terp. - unresolved terpenes mixture.

| **Sherd ID** | **Lot number** | **Site** | **Chronology** | **Weights (g)** | **Period** | **Lipid concentration (μg g-1)** | **δ^13^C_16:0_(^0^/_00_)** | **δ^13^C_18:0_(^0^/_00_)** | **Δ^13^C(^0^/_00_)** | **Assignment** | **Major lipid compounds present** | **Samples that were TMS and run on a high temperature column (HP DB1)** |
| --- | --- | --- | --- | --- | --- | --- | --- | --- | --- | --- | --- | --- |
| 1-I SILV | 11535 | Abri Urschai (Val Urschai, Ftan) | 5^th^ millennium BC | 15.7 | Neolithic | 10.39 | n/a | n/a | n/a | n/a | SFA (C14:0-24:0), UFA (C14:1,C16:1,C18:1,C22:1), br., pri., Alk. (C16-24) |  |
| 2-I SILV | 11536 | Abri Urschai (Val Urschai, Ftan) | 5^th^ millennium BC | 13.54 | Neolithic | 8.8 | -28.07 | -28.82 | -0.75 | Ruminant Carcass Fat | SFA (C12:0-22:0), UFA (C16:1,C18:1,C22:1), br., pri., Alk. (C16-23) |  |
| 3-I SILV | 11537 | Abri Urschai (Val Urschai, Ftan) | 5^th^ millennium BC | 8.02 | Neolithic | 0.38 | n/a | n/a | n/a | n/a | SFA (C12:0-18:0), UFA (C16:1, C18:1), br., Alk. (C16-23) |  |
| 4-I SILV | 11538 | Abri Urschai (Val Urschai, Ftan) | 5^th^ millennium BC | 9.65 | Neolithic | 11.45 | -28.33 | -28.08 | 0.25 | Non-ruminant Carcass fat | SFA (C12:0-24:0), UFA (C16:1, C18:1, C22:1), br., pri., Alk. (C16-26) |  |
| 5-I SILV | 11539 | Abri Urschai (Val Urschai, Ftan) | 5^th^ millennium BC | 11.5 | Neolithic | 579.14 | -28.87 | -29.77 | -0.9 | Rumiant Carcass fat | SFA (C12:0-20:0), UFA (C14:1, C16:1, C18:1, C20:1), br., pri., Alk. (C16-22) |  |
| 40-O-SILV | 13558 | Abri Urschai (Val Urschai, Ftan) | 5^th^ millennium BC | n/a | Neolithic | 148.74 | -28.87 | -30.05 | -1.18 | Rumiant Carcass fat | SFA (C12:0-18:0), UFA (C16:1, C18-1), br., Alk. (C16-22) |  |
| 6-I SILV | 11540 | Plan da Mattun L1 (Val Urschai, Ftan) | Late 2^nd^/Early 1^st^ millennium BC | 9.88 | Bronze Age | 6.74 | -29.6 | -29.29 | 0.31 | Non-ruminant Carcass fat | SFA (C12:0-26:0), UFA (C16:1, C18:1, C22:1), br., pri., Alk. (C16-19) |  |
| 7-I SILV | 11541 | Plan da Mattun L1 (Val Urschai, Ftan) | Late 2^nd^/Early 1^st^ millennium BC | 11.7 | Bronze Age | 17.37 | -28.74 | -28.66 | 0.08 | Non-ruminant Carcass fat | SFA (C12:0-26:0), UFA (16:1, C17:1, C18:1, C22:1), br., pri., Alk. (C16-29) |  |
| 8-I SILV | 11542 | Plan da Mattun L1 (Val Urschai, Ftan) | Late 2^nd^/Early 1^st^ millennium BC | 8.05 | Bronze Age | 12.72 | -28.79 | -29.28 | -0.49 | Mixture of Carcass fat | SFA (C5-26), UFA (C14:1, C16:1, C22:1), DCFA (C9), br., pri., Alk. (C11, C18-27), lact. (γ) |  |
| 9-I SILV | 11543 | Plan da Mattun L1 (Val Urschai, Ftan) | Late 2^nd^/Early 1^st^ millennium BC | 6.63 | Bronze Age | 153.65 | -27.08 | -29.32 | -2.24 | Ruminant Carcass Fat | SFA (C9:0-26:0), UFA (C16:1, C18:1, C22:1), DCFA (C9), br., APFA (C18), phy., pri., Alk. (C12-19), lact. (γ, δ) |  |
| 10-O SILV | 11544 | Plan da Mattun L1 (Val Urschai, Ftan) | Late 2^nd^/Early 1^st^ millennium BC | 4.74 | Bronze Age | 13.72 | -29.02 | -30.23 | -1.22 | Ruminant Carcass Fat | SFA (C12:0-26:0), UFA (C16:1, C18:1, C22:1), br., phy., pri., Alk. (C16-25) |  |
| 11-I SILV | 11545 | Plan da Mattun L1 (Val Urschai, Ftan) | Late 2^nd^/Early 1^st^ millennium BC | 9.27 | Bronze Age | 629.3 | -25.87 | -28.48 | -2.61 | Ruminant Carcass Fat | SFA (C9:0-24:0), UFA (C16:1, C18:1, C22:1), DCFA (C9-17), br., APFA (C16, C18), phy., Alk. (C11-18), lact. (γ, δ) | * |
| 22-I SILV | 11521 | Ils Cuvels (Ova Spin, Zernez) | First half of the 2^nd^ millennium | 13.99 | Bronze Age | 24.23 | -27.8 | -29.32 | -1.52 | Ruminant Carcass Fat | SFA (C12:0-22:0), UFA (C14:1, C16:1, C18:1, C22:1), br., pri., Alk. (C16-24), abiet., |  |
| 24-I SILV | 11523 | Ils Cuvels (Ova Spin, Zernez) | First half of the 2^nd^ millennium | 16.01 | Bronze Age | 52.46 | -26.32 | -27.67 | -1.35 | Ruminant Carcass Fat | SFA (C11:0-26:0), UFA (C16:1, C18:1, C22:1), DCFA (C9), br., pri., Alk. (C16-26), abiet. |  |
| 25-I SILV | 11524 | Ils Cuvels (Ova Spin, Zernez) | First half of the 2^nd^ millennium | 14.07 | Bronze Age | 53.62 | -26.74 | -28.79 | -2.05 | Ruminant Carcass Fat | SFA (C11:0-26:0), UFA (C14:1, C16:1, C17:1, C18:1, C22:1), DCFA (C9), br., pri., Alk. (C16-24), lact. (γ), chol., abiet. |  |
| 26-I SILV | 11525 | Ils Cuvels (Ova Spin, Zernez) | First half of the 2^nd^ millennium | 19.25 | Bronze Age | 103.51 | -28.06 | -29.84 | -1.78 | Ruminant Carcass Fat | SFA (C11-26), UFA (C14:1, C16:1, C17:1, C18:1, C22:1), DCFA (C9), br., pri., Alk. (C15-21), chol., abiet. |  |
| 12-O SILV | 11516 | Iron Age Hut (Val Fenga, Ramosch) | First half of the 1^st^ millennium BC | 2.68 | Iron Age | 810.08 | -27.1 | -32.25 | -5.15 | Dairy | SFA (C8:0-26:0), UFA (C16:1, C18:1), DCFA (C9), br., APFA(C18), phy., Alk. (C12-22), lact. (γ, δ) |  |
| 13-O SILV | 11517 | Iron Age Hut (Val Fenga, Ramosch) | First half of the 1^st^ millennium BC | 6.29 | Iron Age | 980.3 | -27.58 | -32.44 | -4.86 | Dairy | SFA (C9:0-26:0), UFA (C18:1), DCFA (C9), br., APFA (C18), phy., Alk. (C12-22), Ket. (14-C29, 16-C31, 16-C33, 18-C35), lact. (γ, δ) |  |
| 14-I SILV | 11518 | Iron Age Hut (Val Fenga, Ramosch) | First half of the 1^st^ millennium BC | 11.76 | Iron Age | 327.19 | -27.65 | -31.21 | -3.56 | Dairy | SFA (C8:0-28:0), UFA (C18:1), DCFA (C9), br., Alk. (C13-18), lact. (γ), terp. | * |
| 15-I SILV | 11519 | Iron Age Hut (Val Fenga, Ramosch) | First half of the 1^st^ millennium BC | 23.81 | Iron Age | 659.84 | -27.1 | -29.91 | -2.81 | Ruminant Carcass Fat | SFA (C10-26), UFA (C16:1,C18:1,C22:1), DCFA (C9-17), br., Alk. (C13-20), lact. (γ), terp. |  |
| 16-I SILV | 11520 | Iron Age Hut (Val Fenga, Ramosch) | First half of the 1^st^ millennium BC | 17.35 | Iron Age | 1762.97 | -26.55 | -29.71 | -3.16 | Mixed carcass dairy | SFA (C10:0-26:0), UFA (C16:1, C18:1), DCFA (C9-17), br., APFA (C18), Alk. (C13-24), lact. (γ, δ), terp. | * |
| 17-O SILV | 11526 | Plan d’Agl (Val Tasna, Ardez) | Late 2^nd^/1^st^ millennium BC | 4.34 | Late Bronze Age/Iron Age | 235.23 | -27.42 | -29.37 | -1.95 | Rumiant Carcass fat | SFA (C10:0-26:0), UFA (C16:1, C18:1), DCFA (C9-17), br., APFA (C18), phy., Alk. (C14-22), lact. (γ, δ) |  |
| 18-O SILV | 11527 | Plan d’Agl (Val Tasna, Ardez) | Late 2^nd^/1^st^ millennium BC | 4.96 | Late Bronze Age/Iron Age | 63.95 | -27.35 | -31.64 | -4.29 | Dairy | SFA (C12:-26:0), UFA (C16:1, C18:1, C22:1), br., APFA (C18), Alk. (C16-18), terp. |  |
| 19-O SILV | 11528 | Plan d’Agl (Val Tasna, Ardez) | Late 2^nd^/1^st^ millennium BC | 2.02 | Late Bronze Age/Iron Age | 148.27 | -27.02 | -28.64 | -1.62 | Ruminant Carcass Fat | SFA (C12-26), UFA (C16:1, C18:1, C22:1), DCFA (C9-17), br., APFA (C18), phy., Alk. (C15-19), Ket. (14-C29, 16-C31, 16-C33, 18-C35), lact. (γ) |  |
| 20-O-SILV | 11529 | Plan d’Agl (Val Tasna, Ardez) | Late 2^nd^/1^st^ millennium BC | 3.92 | Late Bronze Age/Iron Age | 42.2 | -28.25 | -30.62 | -2.36 | Rumiant Carcass fat | SFA (C14-20), UFA (C16:1, C18:1), br., phy., Alk. (C16-18), lact. (γ) |  |
| 21-O SILV | 11530 | Plan d’Agl (Val Tasna, Ardez) | Late 2^nd^/1^st^ millennium BC | 4.29 | Late Bronze Age/Iron Age | 121.21 | -27.11 | -30.75 | -3.64 | Dairy | SFA (C12-26), UFA (C16:1, C18:1), br., APFA (C18), phy., pri., Alk. (C15-18) |  |
| 27-O SILV | 11531 | Chamanna dal Paster (Val Languard, Pontesina) | First half of the 1^st^ millennium BC | 6.23 | Iron Age | 158.67 | -26.33 | -30.05 | -3.72 | Dairy | SFA (C10:0-28:0), UFA (C16:1, C18:1, C22:1), DCFA (C8-17), br., APFA (C18), phy., pri, Alk. (C13-18), lact. (γ, δ) |  |
| 28-I SILV | 11532 | Chamanna dal Paster (Val Languard, Pontesina) | First half of the 1^st^ millennium BC | 10.58 | Iron Age | 760.36 | -26.92 | -29.74 | -2.82 | Rumiant Carcass fat | SFA (C9:0-26:0), DCFA (C9-17), br., Alk. (C12-18), lact. (γ, δ) | * |
| 29-I SILV | 11533 | Chamanna dal Paster (Val Languard, Pontesina) | First half of the 1^st^ millennium BC | 8.23 | Iron Age | 542.52 | -26.6 | -29.55 | -2.95 | Rumiant Carcass fat | SFA (C9-25), UFA (C22:1), DCFA (C9-17), br., APFA (C18), phy., Alk. (C12-22), Ket. (16-C31, 16-C33, 18-C35), lact. (γ, δ) |  |
| 30-O SILV | 11534 | Chamanna dal Paster (Val Languard, Pontesina) | First half of the 1^st^ millennium BC | 5.47 | Iron Age | 1221.23 | -27.06 | -31.28 | -4.22 | Dairy | SFA (C7-26:0), UFA (C18:1), DCFA (C9-17), br., pri., Alk. (C11-18), lact. (γ, δ) |  |
