# Supplementary material for: Chemical Analysis of Pottery Demonstrates Prehistoric Origin for High-Altitude Alpine Dairying
Source: PLoS One. 2016 Apr 21;11(4):e0151442. doi: 10.1371/journal.pone.0151442 (PMC4839595; doi:10.1371/journal.pone.0151442)
Supplement: S2 Table — (DOCX) [file pone.0151442.s004.docx]

**S2 Table. Summary of GC-C-IRMS analysis of faunal remains**

| **Sample ID** | **Description** | **Category** | **Sample type** | **Provenance** | **δ^13^C_16:0_(^0^/_00_)** | **δ^13^C_18:0_(^0^/_00_)** | **Δ^13^C(^0^/_00_)** |
| --- | --- | --- | --- | --- | --- | --- | --- |
| 37-SILV | Fresh cattle cheese | Dairy |  | Val Tasna, Grisons, CH | -28.2 | -33.5 | -5.2 |
| 38-SILV | Goat milk | Dairy |  | Val Tasna, Grisons, CH | -28.2 | -34.1 | -5.9 |
| 39-SILV | Goat cheese | Dairy |  | Val Tasna, Grisons, CH | -27.6 | -32.3 | -4.8 |
| 32-SILV | Chamois  (*Rupicapra rupicapra*) | Ruminant | Soft tissue | Val Tasna, Grisons, CH | -28.4 | -29.2 | -0.8 |
| 34-SILV | Alpine ibex  (*Capra ibex*) | Ruminant | Soft tissue | Val Tasna, Grisons, CH | -28.1 | -31.0 | -2.8 |
| 42-SILV | White alpine sheep  (*Ovis aries*) | Ruminant | Soft tissue | Val Tasna, Grisons, CH | -28.9 | -31.3 | -2.4 |
| 33-SILV | Roe deer  (*Capreolus capreolus*) | Ruminant | Soft tissue | Val Tasna, Grisons, CH | -29.6 | -30.7 | -1.2 |
| 36-SILV | Red deer  (*Cervus elaphus*) | Ruminant | Soft tissue | Val Tasna, Grisons, CH | -29.7 | -34.0 | -4.2 |
| 31-SILV | Chamois  (*Rupicapra rupicapra*) | Ruminant | Soft tissue | Val Tasna, Grisons, CH | -27.8 | -30.2 | -2.4 |
| 43-SILV | White alpine sheep  (*Ovis aries*) | Ruminant | Soft tissue | Val Tasna, Grisons, CH | -30.2 | -31.6 | -1.5 |
| 44-SILV | Reathian grey cattle  (*Bos taurus*) | Ruminant | Soft tissue | Val Tasna, Grisons, CH | -29.1 | -32.1 | -3.0 |
| 35-SILV | Alpine marmot  (*Marmota marmota*) | Non-ruminant | Soft tissue | Val Tasna, Grisons, CH | -30.4 | -29.9 | 0.4 |
