# Supplementary material for: Chemical Analysis of Pottery Demonstrates Prehistoric Origin for High-Altitude Alpine Dairying
Source: PLoS One. 2016 Apr 21;11(4):e0151442. doi: 10.1371/journal.pone.0151442 (PMC4839595; doi:10.1371/journal.pone.0151442)
Supplement: S3 Table — (DOCX) [file pone.0151442.s005.docx]

**S3 Table. Radiocarbon dates from the sites associated with pottery analysed**

| **Site** | **Sample** | **Date (yrs BP)** | **Date (yrs BC)**  **Cal 2 sigma** |
| --- | --- | --- | --- |
| Abri Urschai (Val Urschai, Ftan) | ETH-58323 | 5346+-30 | 4320-4050 |
|  | ETH-58324 | 5303+-31 | 4240-4040 |
|  | ETH-58325 | 5740+-31 | 4690-4500 |
|  | ETH-59917 | 5782+-33 | 4712-4546 |
|  | ETH-59919 | 5787+-33 | 4714-4548 |
|  | ETH-59920 | 5395+-34 | 4339-4076 |
|  | ETH-59921 | 5374+-33 | 4331-4066 |
|  | ETH-59922 | 5870+-34 | 4834-4620 |
|  | ETH-59923 | 5430+-34 | 4346-4236 |
|  | ETH-59924 | 5427+-28 | 4340-4242 |
|  | ETH-59925 | 5538+-28 | 4448-4340 |
|  | ETH-59926 | 5668+-28 | 4558-4448 |
|  | ETH-59927 | 5333+-28 | 4256-4051 |
|  | ETH-59928 | 5402+-28 | 4336-4178 |
| Ils Cuvels (Ova Spin, Zernez) | ETH-58326 | 3386+-28 | 1750 -1620 |
|  | ETH-58328 | 3494+-29 | 1900-1700 |
| Plan da Mattun L1 (Val Urschai, Ftan) | ETH-39646 | 2790 ±40 | 1040-830 |
|  | ETH-39645 | 2535 ±45 | 900-780 |
| Iron Age Hut (Val Fenga, Ramosch) | ETH-34341 | 2425 ±55 | 670-390 |
|  | ETH-36465 | 2535 ±45 | 810-510 |
|  | ETH-59893 | 2729+-28 | 923-815 |
|  | ETH-59894 | 2522+-25 | 792-546 |
|  | ETH-59895 | 2495+-29 | 781-536 |
|  | ETH-59898 | 2801+-30 | 1026-850 |
|  | ETH-59899 | 2530±29 | 796-544 |
|  | ETH-59900 | 2633±29 | 840-780 |
| Chamanna dal Paster (Val Languard, Pontesina) | ETH 36723 | 2505+-35 | 800-510 |
| Plan d’Agl (Ardez, Val Tasna) | ETH-36464 | 2210 ±45 | 390-170 |
|  | ETH-34479 | 2190 ±50 | 390-110 |
|  | ETH-59890 | 2882 ±29 | 1192-942 |
|  | ETH-59892 | 2587±29 | 816-754 |
