# Supplementary material for: Chemical Analysis of Pottery Demonstrates Prehistoric Origin for High-Altitude Alpine Dairying
Source: PLoS One. 2016 Apr 21;11(4):e0151442. doi: 10.1371/journal.pone.0151442 (PMC4839595; doi:10.1371/journal.pone.0151442)
Supplement: S1 Text — (DOCX) [file pone.0151442.s006.docx]

**S1 Text**

**Geographical setting**

Four of the investigated sites are located in the Silvretta mountain range, in Lower Engadin, along the Austrian-Swiss border [[1]](https://paperpile.com/c/p4naIe/xa9pP). The Ils Cuvels site is located in the Livigno mountain range, about 12 km south of the Silvretta massif. The Chamanna dal Paster site is further south-east, at the junction of the Upper Engadin with the Bernina Valley (Bernina mountain range). All of these areas belong to the so-called Western Rhaetian Alps, and are characterized by metamorphic geology [[2]](https://paperpile.com/c/p4naIe/LsFaY). The Engadin valley bottom is 1,000 to 1,800 m asl, and the highest peaks that surround it are well above 3,000 m asl, with the Piz Bernina reaching 4,048.6 m. Pastures along and above the current timberline (about 2,000 m asl) are still exploited for livestock grazing (mainly cattle, sheep, and goats) throughout the summer season [[3]](https://paperpile.com/c/p4naIe/ceQY1). The climate at high altitude can be described as cold and wet. In the Silvretta valleys, for instance, the mean annual precipitation is 900 mm on the northern side and 695 mm on the southern side, and the annual mean temperatures are 3 to 5^o^C [[4]](https://paperpile.com/c/p4naIe/DcvHJ). Human impact on the local environment is recorded since the 5^th^ millennium BC [[5,6]](https://paperpile.com/c/p4naIe/W8l0O+PbcTM), and it became more intense during the 2^nd^ millennium BC [[7]](https://paperpile.com/c/p4naIe/zEfKe), when the earliest permanent archaeological sites are documented in the lowlands [[8]](https://paperpile.com/c/p4naIe/PUfkn).

**Brief description of the investigated sites**

**Abri Urschai (Val Urschai, Ftan)**; Long 10.20373, Lat 46.83401 – A rock-shelter located near a modern pastoral cabin, at the junction of the Urschai Valley with the Tasna Valley, around 2,180 m asl. A number of fireplaces and small pits were identified and partly excavated under the sheltered area. A quite rich archaeological and faunal assemblage was discovered. Several flint artefacts were identified, including flint arrowheads. Most of the animal bone fragments could not be identified, with a few identified as wild ruminants and rabbit. It was also very difficult to assign the non-decorated potsherds to a recognised chrono-typology. Radiocarbon dating suggested that this rock-shelter was occupied between the 5^th^ and the 3^rd^ millennium BC. The main archaeological and faunal assemblages seemed to be associated to the earliest occupation phase at this site.

**Ils Cuvels (Ova Spin, Zernez)**; Long 10.16049, Lat 46.67156 – A large, cave-like rock-shelter, originally excavated by amateur archaeologists in the 1930s. Several phases of occupation were recorded, including a phase radiocarbon dated to the Early to Middle Bronze Age (first half of the 2^nd^ millennium BC). This phase yielded a significant faunal assemblage (wild and domestic animals), as well as coarse, but partially decorated potsherds. Although this is not strictly a high-altitude site, being located at 1,680 m asl, in the context of this study, we compare and contrast results with the other investigated sites.

**Plan da Mattun L1 (Val Urschai, Ftan)**; Long 10.22727, Lat 46.85116 – A rock-shelter situated at the head of the Urschai Valley (2,287 m asl), an area characterized by the presence of several boulders. Different phases of human occupation were recorded at this site, from the Mesolithic to the Modern times. The Bronze Age phase is particularly well documented: a number of fireplaces were stratigraphically associated with flint objects, bronze artefacts, and pottery fragments (attributed to the Laugen-Melaun group), along with ovi-caprine bones showing traces of slaughtering. Radiocarbon dating and the chronological attribution of the potsherds suggested that this phase can be dated to the end of the 2^nd^ millennium BC and the very beginning of the 1^st^ millennium BC.

**Iron Age Hut (Val Fenga, Ramosch)**; Long 10.25887, Lat 46.90821 – The foundation of a dry-stone cabin (7 x 7 m) was excavated at 2,283 m asl. Calcined bones found within this structure (earth oven) were attributed to ovi-caprines and (mostly) bovines. Fine ware fragments collected during the excavation can be assigned to the Laugen-Melaun group and Taminser Gruppe (Inner Alpine/Rhine valley group) of the Iron Age. Radiocarbon and dendrochronological dates suggest an occupation of this structure around the mid-1^st^ millennium BC, matching the chronological attribution of the ceramics.

**Chamanna dal Paster (Val Languard, Pontesina)**; Long 9.93815, Lat 46.4821 – A rock-shelter located at 2,415 m asl, with a dry-stone wall constructed across the opening. It yielded archaeological data suggesting a long-term occupation (from the Mesolithic to the 20^th^ century). The Iron Age phase (mid-1^st^ century BC) is particularly well documented, although it is not clear whether the dry-stone structure is attributable to this period or to a historical phase of occupation.

**Plan d’Agl (Ardez, Val Tasna)**; Long 10.19885, Lat 46.82935 – A “D” shaped dry-stone enclosure, covering an area of approximately 200 m^2^, interpreted as pastoral pen. It is located in a large flat pasture area still exploited by local herders, not far from the Abri Urschai. Archaeological trenches were excavated in different sectors of the enclosure and they provided material culture attributed to the Late Bronze Age/Early Iron Age through to the Late Iron Age. Several fireplaces were excavated near a small boulder embedded into the enclosure. Radiocarbon dating of the charcoal samples from these fireplaces suggested different periods of occupation of the site: Iron Age, Late Bronze Age and Early Bronze Age. According to these data and the ceramics, the construction of the dry-stone enclosure took place between the Late Bronze Age and the Early Iron Age, while a more ephemeral phase of pastoral activity is inferred for the Early Bronze Age.

**References**

1. [Reitmaier T, editor. Letzte Jäger, erste Hirten. Hochalpine Archäologie in der Silvretta. Chur: Archäologischer Dienst Graubünden; 2012.](http://paperpile.com/b/p4naIe/xa9pP)

2. [Schmid SM, Rück P, Schreurs G. The significance of the Schams nappes for the reconstruction of the paleotectonic and orogenic evolution of the Penninic zone along the NFP-20 East traverse (Grisons, eastern Switzerland). In: Roure F, Heizman P, Polino R, editors. Deep Structure of the Alps. 1990. pp. 263–287.](http://paperpile.com/b/p4naIe/LsFaY)

3. [Weiss R. Das Alpwesen Graubündens. Wirtschaft, Sachkultur, Recht, Älplerarbeit und Älplerleben. Chur: Octopus; 1992.](http://paperpile.com/b/p4naIe/ceQY1)

4. [Bauerochse A, Katenhusen O. Holozäne Landschaftsentwicklung und aktuelle Vegetation im Fimbertal (Val Fenga, Tirol/Graubünden). Phytocoenologia. 1997;27: 353–453.](http://paperpile.com/b/p4naIe/DcvHJ)

5. [Dietre B, Walser C, Lambers K, Reitmaier T, Hajdas I, Haas JN. Palaeoecological evidence for Mesolithic to Medieval climatic change and anthropogenic impact on the Alpine flora and vegetation of the Silvretta Massif (Switzerland/Austria). Quat Int. 2014;353: 3–16.](http://paperpile.com/b/p4naIe/W8l0O)

6. [Zoller H, Erny-Rodmann P, Punchakunnel P. The history of vegetation and land use in the Lower Engadine (Switzerland): pollen record of the last 13000 years. Zernez: Schweizerischen Akademie der Naturwissenschaften; 1996.](http://paperpile.com/b/p4naIe/PbcTM)

7. [Gobet E, Tinner W, Hochuli PA, van Leeuwen JFN, Ammann B. Middle to Late Holocene Vegetation history of the Upper Engadin (Swiss Alps): the role of man and fire. Vegetation History and Archaeobothany. 2003;12: 143–163.](http://paperpile.com/b/p4naIe/zEfKe)

8. [Rageth J. Kleine Urgeschichte Graubündens. Archäologie der Schweiz. 2000;23: 32–46.](http://paperpile.com/b/p4naIe/PUfkn)
